# Safety and Photoprotective Efficacy of a Sunscreen System Based on Grape Pomace (*Vitis vinifera* L.) Phenolics from Winemaking

**DOI:** 10.3390/pharmaceutics12121148

**Published:** 2020-11-27

**Authors:** Alexandra A. Hübner, Fernanda D. Sarruf, Camila A. Oliveira, Alberto V. Neto, Dominique C. H. Fischer, Edna T. M. Kato, Felipe R. Lourenço, André Rolim Baby, Elfriede M. Bacchi

**Affiliations:** 1 School of Pharmaceutical Sciences, University of São Paulo, São Paulo 05508-000, Brazil; alexandra.hubner@usp.br (A.A.H.); milaareias@hotmail.com (C.A.O.); bertovetore@hotmail.com (A.V.N.); domi@usp.br (D.C.H.F.); myiake@usp.br (E.T.M.K.); feliperl@usp.br (F.R.L.); elfriede@usp.br (E.M.B.); 2 IPclin—Institute of Integrated Clinical Research, Jundiai 13200-000, Brazil; fernanda.sarruf@usp.br

**Keywords:** grape pomace, *Vitis vinifera* L., *Cabernet Sauvignon*, phenolic, antioxidant activity, sun protector factor, SPF, clinical effectiveness, human repeated insult patch test, clinical trial

## Abstract

In winemaking, a large amount of grape pomace is produced that is rich in polyphenolics and highly beneficial for human health, as phenols are useful for skin ultraviolet (UV) protection. In this investigation, we evaluated the safety and clinical efficacy of a sunscreen system containing a grape pomace extract from *Vitis vinifera* L. as a bioactive ingredient. The recovery of phenolics in the waste was performed by percolation. Nine emulsions were developed using a factorial design and two were evaluated clinically: Formulation E, containing only UV filters (butylmethoxydibenzoyl methane, ethylhexyl methoxycinnamate and ethylhexyl dimethyl PABA), and F, with the extract at 10.0% *w*/*w* + UV filters. The antioxidant activity was determined by the DPPH assay and the in vitro efficacy was established by sun protection factor (SPF) measurements (Labsphere UV-2000S). Clinical tests were performed to determine safety (human repeated insult patch test) and to confirm efficacy (photoprotective effectiveness in participants). The results showed a synergistic effect between the sunscreen system and the extract on UVB protection and antioxidant activity. Both samples were considered safe. Formulation F was 20.59% more efficient in protecting skin against UVB radiation, taking approximately 21% more time to induce erythema compared to the extract-free sample.

## 1. Introduction

Over time, functional and morphological changes in the skin are inevitable, increasing the susceptibility to viral infections, carcinogenic processes [1], and hyperpigmentation [2]. As the efficiency of endogenous antioxidants decreases, reactive species induce systemic damage [3]. In addition, environmental factors can accelerate natural skin aging and cell proliferation. UVA radiation degrades the extracellular matrix of the collagen and elastin fibers in the dermis, leaving the fibroblasts less active and favoring the appearance of fine lines and deep wrinkles. UVB radiation is involved in the direct effects on DNA and the induction of matrix metalloproteinase 1 expression [4].

Photoprotection can delay or prevent precancerous lesions and UVB-induced immunosuppression in human skin. Sunscreens minimize damage through physical and chemical mechanisms. Broad spectrum filters are powerful strategies against UVA and UVB protection. Thus, with this goal, many sunscreens incorporate different UV filters into a single product. The active ingredients ethylhexyl methoxycinnamate, ethylhexyl dimethyl PABA, and butylmethoxydibenzoyl methane are efficient UVB and UVA absorbers, but they may suffer physical-chemical interactions, photodegradation, and consequent loss of UV protection. The butylmethoxydibenzoyl methane [5] is 50% less absorbent 1 h after UV irradiation [6].

**Figure 1:**
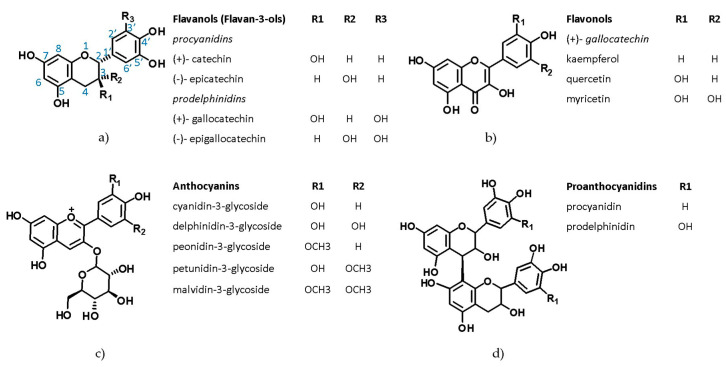
The chemical structures of some groups of polyphenols identified in *V. vinifera* L. grape pomace. Flavonoids (**a**) flavanols (flavan-3-ols); (**b**) flavonols; (**c**) anthocyanins and Condensed tannins; (**d**) proanthocyanidins.

Currently, to optimize sun protection and photostability, sunscreens use natural antioxidant composition [7,8]. In fact, scientific evidence has shown benefits of the topical and oral use of polyphenols from some plant species against UV radiation [9], including *Theobroma cacao* L. [10], *Vitis vinifera* L. [11], *Camellia sinensis* (L.) Kuntze [12,13], *Silybum marianum* (L.) Gaertn. [14], and *Bauhinia microstachya* (Raddi) J.F.Macbr. [13,15]. Figure 1 shows the main phenolic groups identified by our research group for a specimen of grape pomace from *V. vinifera* [16]. The literature attributes the antioxidant activities of *V. vinifera* to fruit skin, with 90% due to anthocyanins and proanthocyanins and 10% to flavonols, flavanols, and phenolic acids. In vivo and in vitro studies of grape seeds attribute the antioxidant action to flavonoids [17]. This grape species is the more common in winemaking [18] and because winemaking waste retains appreciable amounts of polyphenolics [19,20], it could be useful in multifunctional cosmetics.

In 2018, Brazil produced 1,592,242 tons of grapes, of which 818,290 tons were destined to become wine, juice, and derivatives [21]. Furthermore, of 20% of the organic waste generated during winemaking, approximately 16% is grape pomace [18]. Despite the increasing consumption of Brazilian wine, the annual volume (2.2 L per person per year) is lower than that in France (45.7 L per person per year) [22]. This study investigates the safety and photoprotection of cosmetic formulations containing a mixture of the different concentrations of chemical filters: butylmethoxydibenzoyl methane, ethylhexyl methoxycinnamate, ethylhexyl dimethyl PABA, and dry grape pomace extract. The response surface methodology (RSM) was applied to the development of cosmetic preparations and optimization of their variables [23]. In vitro, the sun protection factor (SPF), UVA-PF, and photostability were measured by diffuse reflectance spectrophotometry with an integration sphere [24,25]; and the safety and efficacy of clinical trials were also evaluated [26,27].

## 2. Materials and Methods 

### 2.1. Chemicals

The ingredients used in the photoprotective formulations were ammonium acryloyldimethyltaurate/VP copolymer, trilaureth-4 phosphate, rapeseed oil sorbitol esters, mineral oil and isopropyl palmitate and butylmethoxydibenzoyl methane (PharmaSpecial, Itapevi, SP, Brazil); ethylhexyl methoxycinnamate and ethylhexyl dimethyl PABA methoxycinnamate (Fragon, São Paulo, SP, Brazil); propylene glycol and butyl hydroxy toluene (BHT) (Pharma Nostra, Rio de Janeiro, RJ, Brazil); disodium EDTA and citric acid (All Chemistry do Brasil, Jabaquara, SP, Brazil); sodium hydroxide (Synth, Diadema, SP, Brazil); and phenoxyethanol, methylparaben, ethylparaben and butylparaben (Clariant, Suzano, SP, Brazil). The following reagents and solvents were used: (±)-6-hydroxy-2,5,7,8-tetramethylchromane-2-carboxylic acid-Trolox (Sigma-Aldrich, Steinheim, NW, Germany); methyl alcohol p.a., ACS reagent, 100% (Synth); absolute ethyl alcohol 99.5% (LSChemicals, Ribeirão Preto, SP, Brazil) and purified water (Gehaka equipment, São Paulo, SP, Brazil). The substances were used as received, without any further purification, except for the ethanol that was previously distilled.

### 2.2. Crude Extract

*V. vinifera* var. *Cabernet Sauvignon* cultivated in Southern Brazil was used for winemaking by Beraldo Di Cale Winery in Jundiai, São Paulo. The dried byproduct consisted of 56.24% (*w*/*w*) of skins, 41.57% (*w*/*w*) of seeds, and 2.19% (*w*/*w*) of rachis, pedicels and peduncles (stem sections of the cluster), as determined experimentally in a 150 g sample. The dried plant material was deposited at the Herbarium of the Institute of Biosciences of USP/SP, with the identification of Hübner, No. A1. The active compounds of the by-product were extracted by our research group following the methodology of the Brazilian Pharmacopoeia, 5th edition [28] and Hubner and collaborators [16]. The hydroethanolic extract 70% (*v*/*v*) was prepared from grape pomace (3.2 kg) dried and pulverized by the percolation process until the drug is exhausted. The percolate was concentrated in an ascending film evaporator (academic development), homogenized, and lyophilized (Lyophilizer model Liotop K202). The dried extract was used in in vitro and clinical experiments.

### 2.3. Design of Experiment (DoE)

**Figure 2:**
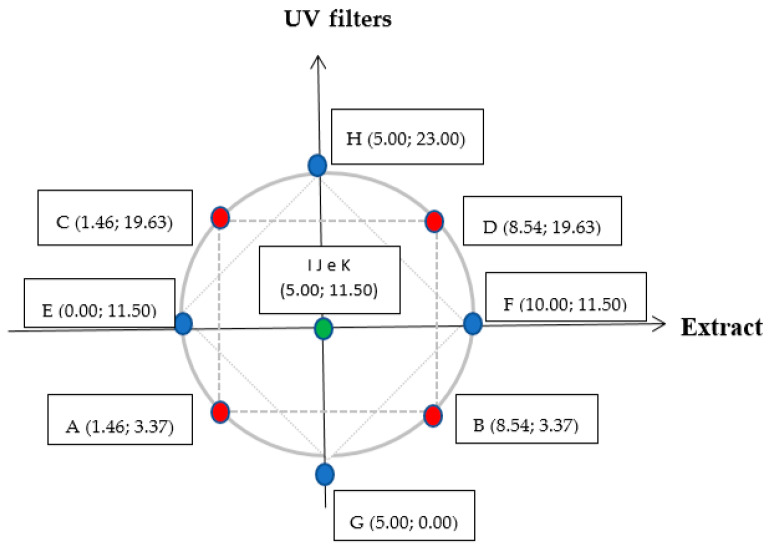
Schematic representation of the DoE using different concentrations (% *w*/*w*) of grape pomace (*V. vinifera* L.) and UV filters. (X, Y)—X is the total concentration of extract in % (*w*/*w*) and Y is the total UV filter concentration in % (*w*/*w*). (**A**–**K**) cosmetic formulations. Extract: dried red grape pomace. UV filters: butylmethoxydibenzoyl methane (UVA), ethylhexyl methoxycinnamate (UVB) and ethylhexyl dimethyl PABA (UVB).

The response surface methodology was used to evaluate the effect of independent variables such as the concentration of UV filters, the extract concentration and irradiation time dependent on the SPF in vitro response, antioxidant activity, critical wavelength (nm), UVA and UVB, and UVA/UVB ratio. The central composite design (CCD) included three central points (green), 4 factorial scores (red) and 4 axial points (blue), as shown in Figure 2.

#### 2.3.1. Formulations

The development of *oil*-in-*water* (*o*/*w*) emulsions followed the factorial design with 2 factor points, 3 axial points, and 3 central points, totaling 11 evaluable samples (A, B, C, D, E, F, G, H, I and K), as shown in Table 1. The aqueous phase was homogenized in Ultra-Turrax (*IKA* T25, Staufen, BW, Germany) in 4 cycles of 5 min at a rotational speed of 8000 rpm and subsequently heated to 50 °C. The ingredients of the oily phase were solubilized at 65–70 °C. The two phases were mixed at ± 40 °C with the aid of a mechanical stirrer (IKA RW 20.n, Burladingen, BW, Germany) for 10 min at 1000 rpm. Finally, the preservative and pH regulator were added to the samples. The preparation and analysis of each sample was performed in triplicate [27,28,29]. The organoleptic properties of the formulations were evaluated by aspect, color, and odor. The pH value was obtained from dispersions of the samples in distilled water (1:10). Sample analysis was performed using a pH meter model (Quimis G400, Diadema, SP, Brazil) [29,30].

#### 2.3.2. Antioxidant Activity

The antioxidant activity was measured according to Brand-Williams; Cuvelier; Berset [31], Hübner and collaborators [16] and Peres and collaborators [6] methods. Parameters by RSM was used to evaluate the SPF, critical wavelength, UVA and UVB protection, and the UVA/UVB ratio was a function of the independent variables (extract, UV filters, and irradiation time). Aliquots of the formulations (1.0% *w*/*w*) were solubilized in methanol and sonicated for 20 min, and the samples were adjusted with pH values close to 5. In test tubes, 1.0 mL of each diluted formulation was mixed to 3.9 mL of 70 μM 2,2-diphenyl-1-picrylhydrazyl (DPPH) methanolic solution. The samples were spectrophotometrically evaluated (Thermo Scientific Evolution 600 UV-Vis, Madison, WI, USA). The antioxidant capacity was expressed in Trolox equivalents (μmol TE g-1) by constructing a standard curve in the following concentrations: 3.12; 25.0; 50.0; 100.0; 125.0; 150.0; 200.0 and 250.0 μg mL^−1^ (R^2^ = 0.9986). The DPPH inhibition percent, as the mean ± standard deviation, was calculated from the triplicate.

#### 2.3.3. Sun Protection Factor (In Vitro SPF) and Photostability

In vitro SPF and the photostability of the formulations in Table 1 were performed according to the method described by Hübner and collaborators [16]. The preparations were weighed and uniformly applied on polymethylmethacrylate (PMMA) plaques (HelioScreen, North Sutton, NH, USA) at the ratio of 0.75 mg·cm^2^ and incubated in darkness for 20 min at room temperature. Each sample was evaluated in the wavelength range of 250–450 nm by diffuse reflectance spectrophotometry with an integrating sphere (Labsphere UV-2000S, North Sutton, NH, USA), with 7 different points of reading and coefficient of variation (COV) < 20 [16]. Next, the same sample plates were irradiated for 1 and 2 h in the solar simulator (Atlas Suntest CPS+, Linsengericht, Hesse, Germany), with a xenon lamp, irradiation dose at 500 Wm^−2^ and temperature of 35 °C. The measurements were performed in triplicate and the mean transmittance values [%] pre and post irradiation were used to calculate SPF, critical wavelength value (λc), UVA and UVB radiation and UVB/UVA ratio by Labsphere software [24,25,32,33]. 

### 2.4. Clinical Trials

#### 2.4.1. Subjects

The Research Ethics Committee of the Faculty of Pharmaceutical Sciences of the University of São Paulo approved the clinical trials under the Certificate of Presentation to Ethics Appreciation (CAAE) n° 46383115.0.3001.8021 and following the norms of the Declaration of Helsinki [34]. Exclusion criteria for the subjects were pregnancy or lactation, history of previous allergic reaction, skin cancer, or dermatological problems in the tested area. If any volunteer showed allergic signs at the negative control site, he would also be excluded from the survey. The inclusion criteria were female or male, phototype I to III, age between 18 and 65 years, and presenting with healthy skin in the back region. Participants were followed directly at all stages of the study, including one month after the tests, by the dermatologist and university researchers [24,35,36].

#### 2.4.2. *Primary and Cumulative Cutaneous Irritability and Sensitization Tests*

These tests included sixty male and female subjects from 18 to 64 years old and with skin phototypes I to IV [37]. The study lasted 6 weeks and was divided into three stages:

- Induction (1st to 3rd week): hypoallergenic adhesive tape (Finn Chambers, Epitest, Rannankoukku, Tuusula, Finland) containing the test samples (E and F) and distilled water as negative control was applied at random sites on the back of each volunteer. If any volunteer showed signs at this negative control site, they would have been excluded from the research. Clinical signs and discomfort were reported to the dermatologist and the samples were reapplied in the same area;
- Rest (4th to 5th week): no product application;
- Challenge (6th week): the samples were applied and remained in contact with the skin for 48 h. The adhesive tape was removed from the skin site and after 30 min the treated areas were clinically evaluated [27,34].

#### 2.4.3. Phototoxicity and Photosensitization Test

In the phototoxicity and photosensitization test, thirty male and female subjects from 18 to 64 years old and with phototypes I to IV were enrolled [37]. The study lasted five weeks and was divided into three stages:

- Induction (1st to 2nd week): hypoallergenic adhesive tape (Finn Chambers, Epitest) containing the samples (E and F) and distilled water as negative control was randomly applied on the back skin of the subject and maintained for 48 h, after which the adhesive tape was removed from the skin and the areas were exposed to UVA and UVB radiation for 6 min 4 s. Clinical signs were reported to the dermatologist and products were reapplied in the same area;
- Rest (3rd to 4th week): no sample was applied;
- Challenge (5th week): samples were in contact with the skin for 48 h, after which the adhesive tape was removed from the skin and the areas were re-irradiated.

Clinical evaluation was performed after 30 min of irradiation. An artificial UVA solar simulator (ProLumina, Cotia, SP, Brazil) of 100 Watts was used, with digital control of the irradiation time. The radiation was treated by an UV radiation meter (Solarmeter, Glenside, PA, USA) and a lamp with intensity of 10 mW·cm^−2^ [27,34].

#### 2.4.4. Clinical Photoprotective Effectiveness 

The clinical photoprotective effectiveness was based on the international sun protection factor test [36]. The SPF of the samples E and F was calculated by the following Equation (1) [34]:

***SPF*** = MED p/MED np (1)

where the Sun Protector factor (***SPF***) is the ratio Minimal Erythema Dose of UV radiation to produce erythema with definite visible edges on protected skin (MED p) and unprotected skin (MED np) with sunscreen.

Ten individuals between 37–61 years of age phototypes II and III (Fitzpatrick, 1975) were enrolled in the study. On the first day, the participants were dermatologically evaluated and samples E and F and the reference product P2 (SPF 16) were applied uniformly (2.0 mg·cm^−2^) at random sites in the dorsal area between the scapulae and hip, previously demarcated in 35 cm^2^, in each volunteer. After 15 min of drying, the areas were irradiated with an artificial solar simulator (Solar Light, Multiport 601, Glenside, PA, USA) with emission in the UVA and UVB bands. Subsequently, 20 ± 4 h after the test, MED p and MED np were observed and the SPF values of the samples were calculated [34].

#### 2.4.5. UVA Protection Factor (UVA-PF) 

In vitro, UVA-PF of formulations E and F were analyzed followed the International Sun Protection Factor Test specifications [25] using a diffractive reflectance spectrometer with integration sphere (Labsphere UV-2000S, North Sutton, NH, USA) and photoprotective efficacy values from the clinical test. Each sample was applied over PMMA plates (HelioScreen, North Sutton, NH, USA), in triplicate, at a ratio of 1.30 mg·cm^−2^ and placed in the dark for 20 min at room temperature. Plates were evaluated at wavelength ranges of 250–450 nm and at least 7 different reading points were assessed. The samples were exposed to artificial sunlight (Atlas Suntest CPS+, Linsengericht, Hesse, Germany) at a dose of ultraviolet radiation of 580.08 W.m^−2^ for 25 min, an experimentally determined period [24]. The mean absorbance values obtained before and after irradiation were used to calculate the UVA-PF by Labsphere software.

### 2.5. Statistical Analysis

The results were evaluated by Minitab 17 statistical software (State College, PA, USA) using multiple comparisons ANOVA and two samples *t*-test and paired *t*-test.

## 3. Results

### 3.1. Design of Experiment (DoE)

The adjusted regression models allowed for the prediction of the influence of independent variables (UV filter amount/concentration (X1), extract concentration (X2) and irradiation time (X3) in response curves for the solar protection factor (Y1), antioxidant activity (Y2), critical wavelength (Y3), UVA transmittance (Y4), UVB transmittance (Y5), and UVA/UVB ratio (Y6). According to the ANOVA results for the regression equation (Table 2 and Table 3), the selected statistical models were adjusted in order to allow the optimization of the independent variables. The adjusted and prediction correlation coefficients were 0.8295 and 0.9907, respectively.

#### 3.1.1. Formulations 

**Figure 3:**
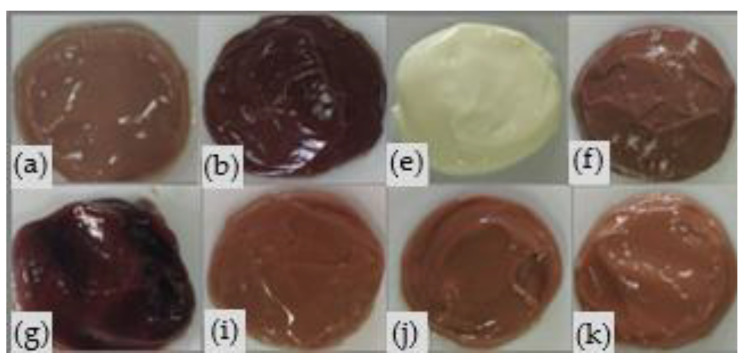
Topical delivery systems developed by the experimental design—DoE (**a**); (**b**); (**e**); (**f**); (**g**); (**i**); (**j**); (**k**) using grape pomace (*V. vinifera* L.) and UV filters (butylmethoxydibenzoyl methane—UVA, ethylhexyl methoxycinnamate and ethylhexyl dimethyl PABA-UVB) and analyzed after 24 h of preparation.

Three points of the DoE (2 factorials (C and D), 1 axial (H)) could not be studied due to problems of stability; i.e., phase separation. Figure 3 shows only the topical delivery systems evaluated in vitro (A, B, E, F, G, I, J, and K) and on the clinical studies (E and F). 

The formulations A, B, E, F, G, I, J, and K (Table 4) were macroscopically stable, with homogeneous aspect, color, and odor characteristics of the raw materials employed. The color intensification of the samples containing extract was observed with the increase in the dried bagasse concentration and the reduction in UV filters.

#### 3.1.2. Antioxidant Activity

**Figure 4:**
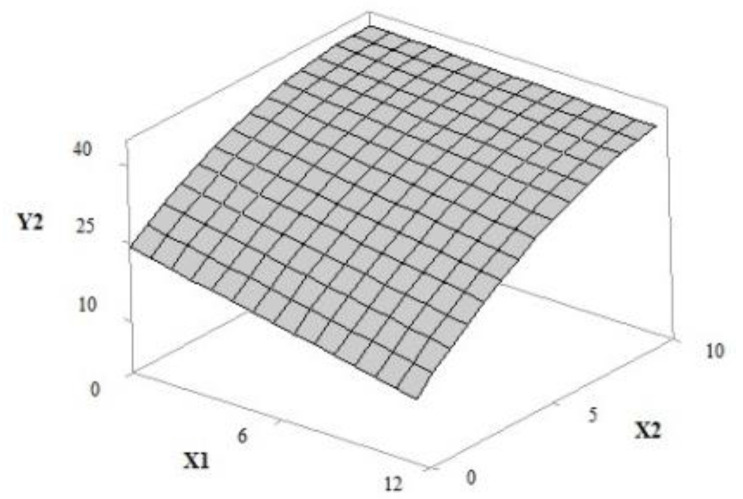
Response surface 3D plot of antioxidant activity—DPPH (Y2) as a function of the concentration of UV filters: butylmethoxydibenzoyl methane—UVA, ethylhexyl methoxycinnamate and ethylhexyl dimethyl PABA—UVB of 0–12% *w*/*w* (X1) and grape pomace extract concentration of 0–10% *w*/*w* (X2).

The surface response of the antioxidant activity showed an inversely proportional relationship between the concentrations of UV filters and extracts (*p*-value, 0.128). The increase in the extract concentration potentiated the antioxidant effect of the samples (*p*-value, 0.010); however, an increase in the concentration of UV filters resulted in a decrease in this parameter (*p*-value, 0.021) (Figure 4).

For the antioxidant activity measured by DPPH inhibition, we observed that formulations B (545.53 ± 0.01 μmol TE g^−1^ grape pomace extract) and F (519.92 ± 0.00 μmol TE g^−1^ grape pomace extract) with the highest contents of grape marc extract (8.54% and 10.0%, respectively) were the best. The antioxidant activity of formulation F was 69.50% higher than that of sample E (64.92 ± 0.00 μmol TE g^−1^ grape pomace extract), without extract and maximum concentration of filters. The results also show a reduction of approximately 20% in the value of antioxidant activity when UV filters (11.50%) were incorporated into the formulations containing 5% of extract (I, J, and K), as showed in Table 5.

#### 3.1.3. Sun Protection Factor (In Vitro SPF) and Photostability

**Figure 5:**
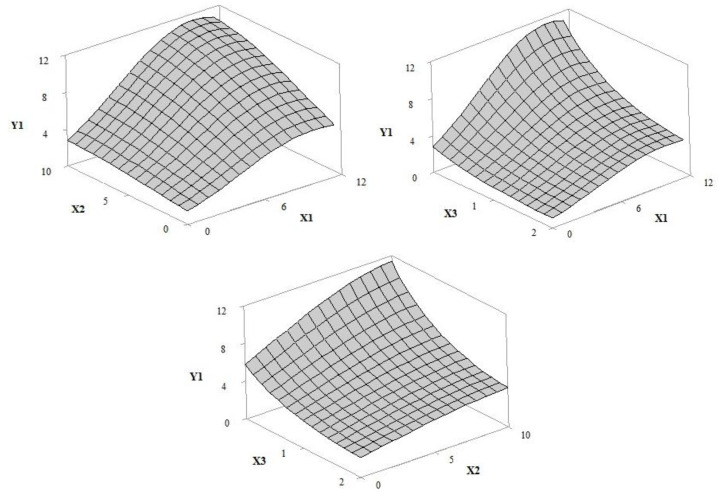
Response surface 3D plot of sun protection factor (Y1) as a function of the concentration of UV filters: butylmethoxydibenzoyl methane—UVA, ethylhexyl methoxycinnamate—UVB and ethylhexyl dimethyl PABA—UVB of 0–12% *w*/*w* (X1), grape pomace extract concentration of 0–10% *w*/*w* (X2) and irradiation time of 1–2 h (X3).

The response surface results shown in Figure 5 revealed an increase in the SPF value in vitro with increased concentrations of UV filters and extract (*p*-values, 0.000 and 0.005, respectively), while the time of exposure to UV radiation reduced this response (*p*-value, 0.000), probably due to the photodegradation of the active compounds. The highest SPF value was observed in formulation F, with 10% grape pomace extract (pre-irradiation SPF 16.33), compared with formulation E, without extract (pre-irradiation SPF 6.00). The sun protection factors of Formulations A and B, with 3.37% of filters each, but with different percentages of extract, 1.46% and 8.54%, respectively, were 4.33 and 5.33.

#### 3.1.4. Critical Wavelength 

**Figure 6:**
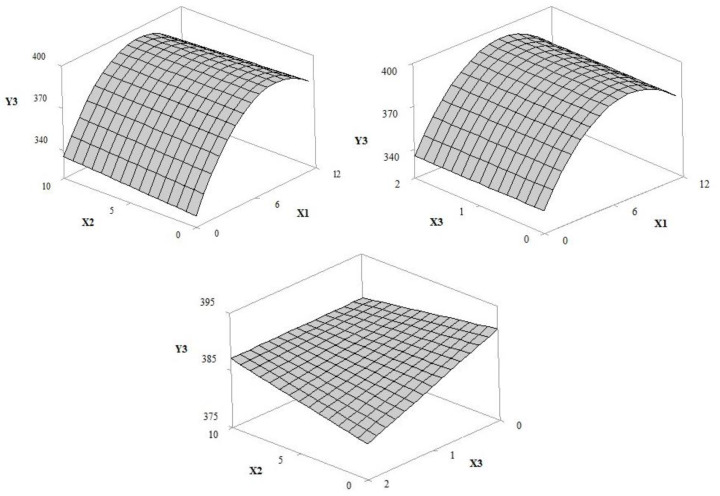
Response surface 3D plot of critical wavelength (Y3) as a function of the concentration of UV filters: butylmethoxydibenzoyl methane—UVA, ethylhexyl methoxycinnamate—UVB and ethylhexyl dimethyl PABA—UVB of 0–12% *w*/*w* (X1), grape pomace extract concentration of 0–10% *w*/*w* (X2) and irradiation time of 1–2 h (X3).

The critical wavelength (λ^crit^) parameter should be greater than 370 nm. In the present study, the pre- and post-irradiation λ^crit^ of formulations E and F were greater than 375 nm; however, there was a decrease when the irradiation time was increased (pre-irradiation 381 nm and 2 h 375 nm; pre-irradiation 379 nm and 2 h: 376 nm, respectively). The surface response graphs in Figure 6 show that the concentration of extracts, UV filters and radiation time had a significant impact on the λ^crit^ (*p*-values, 0.174, 0.000, and 0.0002, respectively). Filters and extract showed a decrease in λ^crit^ as the concentration of these variables decreased (*p*-value, 0.035); however, the ratio between filter concentration and radiation time had no significant effect on this parameter. The central points included the quadratic terms of the regression models, which were relevant to obtain a well-adjusted regression equation. In addition, relative to the exposure time, the extract was inversely proportional, indicating a decrease in the response over time (*p*-value, 0.048).

#### 3.1.5. UVA Transmittance

**Figure 7:**
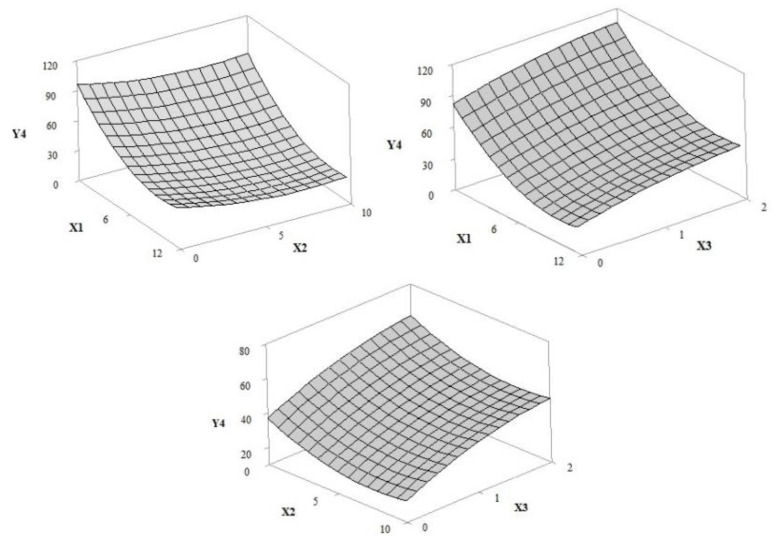
Response surface 3D plot of UVA protection (Y4) as a function of the concentration of UV filters: butylmethoxydibenzoyl methane—UVA, ethylhexyl methoxycinnamate—UVB and ethylhexyl dimethyl PABA—UVB of 0–12% *w*/*w* (X1), grape pomace extract concentration of 0–10% *w*/*w* (X2) and irradiation time of 1–2 h (X3).

According to the surface response graphs in Figure 7, the UVA transmittance was reduced with the increase in extract concentration; thus, there was lower transmission of UVA radiation in samples containing bioactive compounds and filters (*p*-values, 0.009 and 0.000, respectively). However, the response as a function of exposure time increased transmittance and decreased the ability to absorb UVA (*p*-value, 0.003). The lowest percentages of UVA transmittance were observed in the samples with the maximum concentration of UV filters and containing extract (E, F, I, J, and K), or the highest transmittances were observed in the absence or at low concentrations of UV filters (A, B, and G).

#### 3.1.6. UVB Transmittance

**Figure 8:**
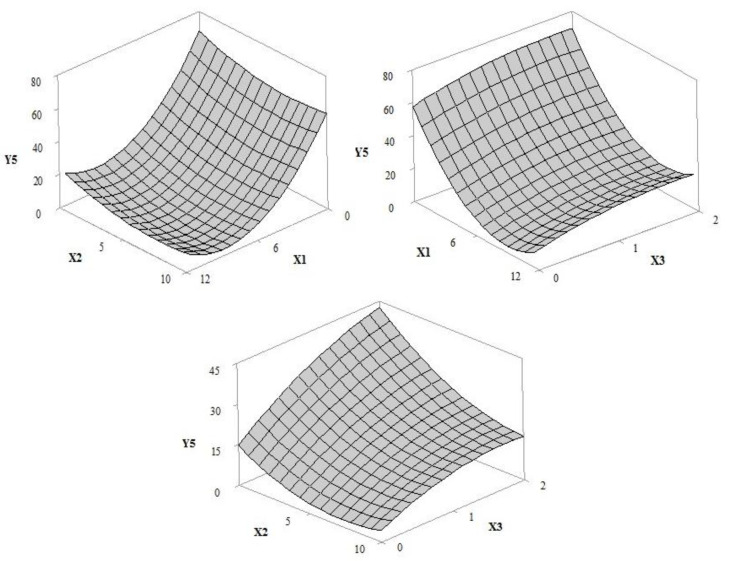
Response surface 3D plot of UVB protection (Y4) as a function of the concentration of UV filters: butyl methoxydibenzoyl methane—UVA, ethylhexyl methoxycinnamate–UVB and ethylhexyl dimethyl PABA—UVB of 0–12% *w*/*w* (X1), grape pomace extract concentration of 0–10% *w*/*w* (X2) and irradiation time of 1–2 h (X3).

Figure 8 shows a decrease in UVB transmittance with the association of ethylhexyl methoxycinnamate at 320 to 290 nm [38,39], ethylhexyl dimethyl PABA at 315 to 290 nm sun filters [40,41] and grape pomace extract (*p*-values, 0.000 and 0.014, respectively). Higher spectral transmission of UVB radiation was observed for formulation G (63.31%) without filters (*p*-value, 0.000). In addition, the irradiation time and the reduction in the concentration of grape pomace extract increased the light transmittance (*p*-value, 0.079). The maximum concentration of UV filters and extract resulted in the lowest percentage of UVB radiation transmitted by formulation F (pre-irradiation 5.44%, one hour 17.54% and two hours 25.37%) compared to formulation E extract-free (pre-irradiation 19.67%, one hour 38.91% and two hours 44.91%). This represents an increase of 14.23% and 19.54% in the absorption of UVB rays before and after two hours of exposure, respectively, compared to that in formulation E. 

#### 3.1.7. UVA/UVB Ratio

**Figure 9:**
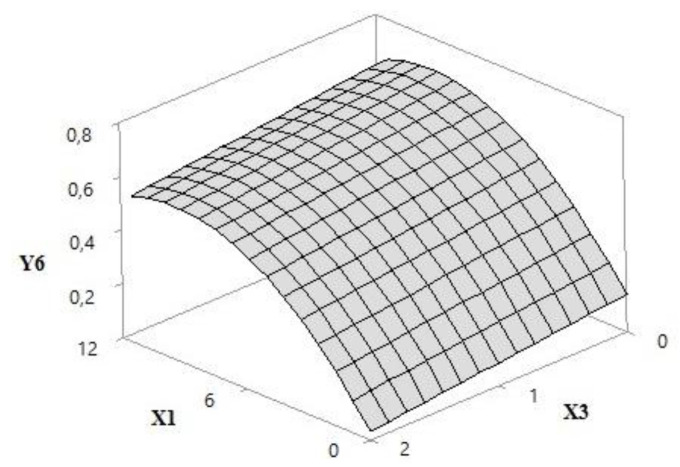
Response surface 3D plot of UVA/UVB ratio (Y6) as a function of the concentration of UV filters: butyl methoxydibenzoyl methane—UVA, ethylhexyl methoxycinnamate—UVB and ethylhexyl dimethyl PABA—UVB of 0–12% *w*/*w* (X1) and irradiation time of 1–2 h (X3).

The response surface in Figure 9 shows a decrease in the in vitro UVA/UVB ratio with the reduction in the UV filter concentration (*p*-value, 0.000). The concentration of the extract had no influence on this parameter. The lowest value was obtained for formulation G (UVA/UVB < 0.20) without synthetic filters. Formulations E, H, I, and J achieved highest ratios and similar responses pre (UVA/UVB > 0.60) and post (UVA/UVB > 0.50) irradiation. After two hours of UV exposure, the mean ratios of the formulations containing 5% grape pomace (I, J and K) were approximately 4% higher than those of the E sample. 

### 3.2. Clinical Trials

#### 3.2.1. Primary, Accumulated Dermal Irritability Test, and Sensitization

The Human Repeated Insult Patch Test (HRIPT) is used to determine the potential of irritation and sensitization of a cutaneous product [42]. In this work, the HRIPT tests performed with the photoprotective formulations did not induce irritability or dermal sensitization.

#### 3.2.2. Phototoxicity and Photosensitization Test

The photoirritant and photosensitizer potential of the topical systems containing grape pomace extract were evaluated by HRIPT, and it did not cause irritability or dermal sensitization.

#### 3.2.3. Photoprotective Effectiveness

**Figure 10:**
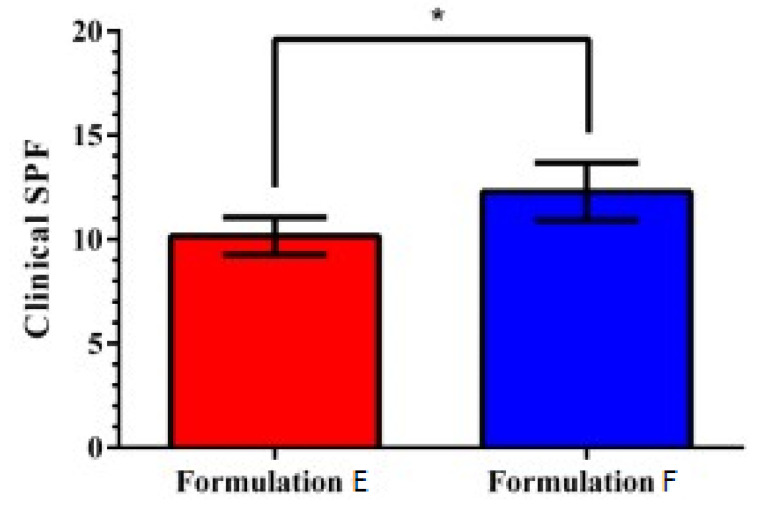
Photoprotective efficacy of formulations (**E**)—Base + UV filters: butylmethoxydibenzoyl methane, ethylhexyl methoxycinnamate and ethylhexyl dimethyl PABA; (**F**)—Base, UV filters and grape pomace extract (*V. vinifera* L.). Paired Student’s *t*-test (*n* = 10): *p*-value, 0.002 * = significant.

Figure 10 shows a superior efficiency in UVB protection of Formulation F with grape pomace compared to E, without extract, as shown by the SPF value of 12.30 and 10.20, respectively. This meant that the formulation F took approximately 21% more time to induce erythema (*p*-value, 0.0264, *n* = 10) compared to sunscreen without extract. 

#### 3.2.4. UVA Protection Factor (UVA-PF)

Formulations E (without grape pomace extract) and F (with extract) were statistically significant in the UVA-PF values (*p*-value, 0.03). The two sunscreens showed values greater than 1/3 of the determined photoprotective efficacy (E = 3.67 and F = 4.33).

## 4. Discussion

Changes in organoleptic attributes such as color and consistency were observed in the evaluated samples with the increased concentration of extract (Figure 3), a parameter generally related to the physical stability of the system [43]. This could have occurred due to the hygroscopic characteristics of the plant material with numerous hydrophilic sites [44].

Phenolic substances can act as antioxidant and metal chelating, inactivating biomolecules by electron/proton transfer from phenols to free radicals [45,46,47]. The antioxidant capacity of the phenolic compounds depends on chemical structure, substituent groups on the aromatic rings A and B [48,49] and hydroxyl standards. The hydroxyl group at the 4 position is more active than those at the 1–2 positions (ortho-meta), as heterocyclic oxygen molecules in the ortho and/or meta position undergo radical stabilization through resonance [49]. For these reasons, natural antioxidants such as tannins, anthocyanins, flavonoids, and phenolic acids present in plant extracts are widely used as additives in foods, beverages, medicines, and cosmetics to free radical scavenging and sequestration and to prevent oxidation [45]. The antioxidant properties of flavonoids are linked to (a) suppression and elimination of reactive oxygen species; (b) inhibition of lipid peroxidation; and (c) regulation of endogenous antioxidant enzymes. Procyanidins participate in the regulation of endogenous antioxidant enzymes and inhibition of lipid peroxidation [50]. Therefore, polyphenolics present in herbal extracts could behave like UV filters, enhancing UV absorption, and reducing the interactions between biomolecules that promote negative changes in the skin [51]. In this study, the intensification of antioxidant activity was correlated with the phenolic content of the extract (E and F). However, a reduction of approximately 20% in the formulation activity (I, J, and K) was detected (Table 5) compared to that of formulation F.

In previous work, chemical filters were found to be fundamental in anti-UVB protection; nevertheless, the addition of pomace meaningfully improved the performance of this factor because of the synergism of phenolics from grape pomace and sun filters [16]. In another study, the *Olea europaea* [52] and *Moringa oleifera* Lam. leaf extracts [53] also improved sunscreens by synergism [52]. Napagoda and collaborators [54], studying eleven herbal extracts, observed a relationship between the strong antioxidant properties of natural substances and high UV absorbance, and, consequently, high SPF values. However, our study showed that the extract was not supposed to act as an UV filter and the in vitro potentiation of the photoprotective efficacy of the extract was dependent on the minimum total concentration of UV filters (11.50% *w/w*) in the formulation, in which the antioxidants from grape pomace were able to interact and produce a positive protection response [16]. As the phenolics can strongly absorb UV radiation, hydroxybenzoic acids (protocatechuic acid, vanillic acid, syringic acid, and gallic acid) show an intense absorption band at 280 nm, while hydroxycinnamic acids (p-coumaric acid, caffeic acid, and ferulic acid) show an absorption band at approximately 320 nm. Both the flavanol monomers/Flavan-3-ols (procyanidins and prodelfinidines) and proanthocyanidins absorb in the range of 280 nm, despite the degree of polymerization [55,56]. The anthocyanins absorb in the visible range at 520 nm; however, when they are acylated with caffeic or p-coumaric acids, they can absorb at approximately 320 nm [56]. Several of the phenolic substances mentioned above have been identified in grape pomace and could be considered as an adjuvant to increase the SPF [16].

Filters must be photostable and efficiently dissipate the incident energy while avoiding the formation of harmful reactive species. To obtain broad-spectrum protection (UVA and UVB protection) and to avoid the instability of chemical UV filters, a commonly used strategy is to combine different filters in low concentrations. This blend can increase safety, optimize synergistic effects with increase in SPF value of the sunscreen, and minimize the instability of UV absorbers. Butyl methoxydibenzoylmethane or avobenzone can benefit from this association; despite being a good absorber in the UVA spectrum range, it is very photo-unstable. It can decrease its photoprotective capacity and even become a reactive photoproduct. Another possibility is the addition of inorganic filters that act by reflecting and propagating UV rays, thus preventing the interaction of chemical filters with UV rays [57]. The quality of sun protection is associated with product stability. In this research, photoinstability of the formulations was observed after artificial UV light in formulation F with 10% extract (post-irradiation 1-h SPF 5.33 and 2 h SPF 3.33) and formulation E without extract (post-irradiation 1 h SPF 2.33 and 2 h SPF 2.00) (Figure 5). The stabilization of the UVA filter avobenzone [58] does not occur linearly and depends on the antioxidant concentration. The time of UV irradiation interferes notably with the stability of the samples, decreasing the SPF [59]. A hypothesis for reducing the photoprotective activity of samples is the photodegradation of sunscreen by interactions with UV radiation. UV filters were essential in UVA protection, especially the performance of the butylmethoxydibenzoyl methane that absorbs radiation in the 320–400 nm range [40,60].

According to the rating criteria “Boot′s star system” recommended by Diffey [33], the better responses obtained for UVA/UVB ratio were verified in formulation E (0.67), which is considered as good protection, followed by formulations H, I, and J with 5% extract (0.65), also considered to provide good protection, and formulation F with 10% of extract (0.59) with moderate pre-irradiation protection (Table 5 and Figure 9). The in vitro UVA-PF method provides a good correlation with clinical results [25]. The use of *V. vinifera* extract enhanced the UVA-PF by 14.17%. Most likely, hydroxyl substituents and the resonance effect of electrons on the conjugated double bonds of the aromatic rings of phenolic compounds give the molecule greater ability to stabilize free radicals and enhance UVA and UVB protection [55].

Irritation is a stimulus caused by irritants above the normal physiological threshold of the skin, resulting in a mild to severe local or systemic inflammatory process clinically characterized by hyperemia, vesicle formation and edema. Sensitization is a reaction that occurs on the skin or mucosa after application of the product [26,61]. Phototoxicity is a nonimmune response with a latency period of hours or days after UV radiation. Photosensitization is an immune response that can occur in individuals previously sensitized by drugs and after UV radiation [62,63,64]. HRIPT tests performed with photoprotective formulations containing hydroalcoholic extract and tested in 60 volunteers did not induce irritability and dermal sensitization during the study, due to no cutaneous inflammatory reaction at the application sites of the samples. A study with the aqueous extract of *V. vinifera* (1%) in 108 patients did not cause irritation or sensitization, corroborating the results of another study that found that the aqueous extract of grape (10%) at a dose of 0.2 mL in 54 subjects was not irritating or sensitizing. Cosmetic formulations containing *V. vinifera* and evaluated by the HRIPTs test showed no irritating nor sensitizing effects with 10% of the fruit; 0.1% of the juice; 0.5% of the juice extract; and 1% seed extract [65]. A water-in-oil emulsion containing 2% grape seed extract *V. vinifera* var. *Muscat Hamburg* did not cause hypersensitivity [66]. HRIPT with fruits of *V. vinifera* (3% and 6%) did not cause skin irritation and sensitization. Another study carried out on 31 volunteers found that the post-beard formulation containing butylene glycol and *V. vinifera* grape seed extract (0.15%) was not irritating to the skin [65]. Products containing 3–10% of *V. vinifera* fruit extract did not trigger irritation and sensitization effects on the skin of volunteers. The creams developed from grape seed extracts were considered safe and nonirritating to the skin in the Burchard test [67]. Skin treated with grape seed extract before UV exposure showed less p53 mutant cells and more epidermal cells and Langerhans cells compared to untreated skin [68], suggesting greater protection of the human genome against photoaging, mutations, and skin cancer [69]. A study carried out with 22 winegrowers showed a weak positive reaction in six individuals who worked directly on grapevines. However, the reactions did not increase with UV irradiation and decreased by 96 h [70].

*Bauhinia microstachya* var. *massambabensis* Vaz obtained by extraction with different solvents also increased the photoprotective effect of O/W emulsions in human skin, SPF 17.90, and SPF 18.98, compared to the sample without extract—SPF 13.48 [15]. *Merostachys pluriflora* Munro ex E.G stem extract (5%) with commercial sunscreens increased the SPF significantly in human subjects. In addition, the authors suggested that the richness of p-coumaric acid in the sample may have been responsible for the intensification of photoprotective activity [71]. Among the hydroxycinnamic acids, the p-coumaric is one of the most abundant in *V. vinifera* grape pomace [72]. Based on the interaction of UV rays with skin filters/chromophores that stimulate the formation of free radicals [58], natural antioxidants, such as extracts or isolated substances, play an important role against these reactive species [51]. For example, due to their electronic deficiency, anthocyanins can efficiently sequester reactive oxygen species (ROS), such as cyanidin-3-glucoside, that act against UVA and UVB radiation in human keratinocytes (HaCaT) [14]. The antioxidant properties of flavonoids have been shown to protect the skin from UV radiation [14,69] and when associated with titanium dioxide offer UVA protection [73]. The combination of quercetin and rutin (10%) in sunscreens has provided SPF values like homosalate, a synthetic UV filter.

In view of not having found scientific evidence of photoprotective efficacy of *C. Sauvignon* grape pomace in sunscreen, comparisons were difficult to establish. The research showed the topical use of grape pomace in formulations was successful in vitro and in clinical trials, probably due to higher concentration of polyphenolics, the antioxidant activity, the UVA and UVB photoprotective activity, and the delayed time of erythema formation by increasing the SPF (Figure 10). Erythema induced by the short wavelength range (UVB, 290–320 nm) is called sunburn [39,74]. The intensity of the erythema increases with the exposure dose and the inflammatory response depends on the wavelength and the penetrating power of UV radiation into the skin. UVC light is absorbed in the epidermis, UVB mainly in the upper dermis, and UVA in the deep dermis. The formation of erythema by UVB can take seconds to 24 h and can disappear in 72 h. Interactions between the UV spectrum and the skin′s chromophores induce the production of reactive oxygen species (ROS), affect the DNA, and induce biochemical and immunological changes. The metabolites of arachidonic acid, inflammatory cytokines, adhesion molecules, and mediators derived from master cells also play a role in the inflammatory pattern [74]. After sun exposure, there may be activation of acute and chronic inflammatory pathways. In this context, antioxidant and anti-inflammatory substances, without the UV filter properties in sunscreen, can also indirectly provide skin photoprotection [75].

The photoprotective efficacy of a sunscreen is based on the criteria for inhibiting cutaneous erythema after exposure to UVB radiation and is classified according to the SPF value. Thus, some sunscreens have in their composition plant extracts with anti-inflammatory activity, such as *Olea europaea*, *C. sinensis*, *Chamomilla recutica* (L.) Rausch, and *Glycyrrhiza glabra* L. to suppress induced erythema by UV and/or increase the SPF. However, the maintenance of the stability of the formulations against UV radiation requires more investigation [76]. Flavonoids, potent natural antioxidants, such as flavonols (quercetin, rutin, kaempferol and myricetin), can have aromatic carbonyl chromophores that are conjugated to the aromatic ring and absorb UVA light between 350–373 nm [77,78,79]; therefore, they are potential sunscreen agents [80]. Rutin (quercetin-3-rutinoside) showed the ability to stabilize UVA filters [81] and the combination of rutin (quercetin-3-O-rutinoside) and quercetin (3′,4′-dihydroxiflavonol) flavonols showed a synergistic effect with a decrease in the transmission of UVA and UVB radiation [73]. The flavonols are usually present in grape pomace extract; thus, we can infer that these polyphenols may improve UVA protection of the photoprotective formulations in this research [55,56,82]. Thus, from a chemical point of view, a plant extract is a complex combination of various substances, so one cannot affirm the role of each substance in the biological effect, without first isolating it and testing it, given the fact that synergism is common in this type of material.

## 5. Conclusions

The cosmetic formulations were optimized by the experimental design. The best efficiency response was found for Formulation F (with 10% *w*/*w* grape pomace extract and 11.5% *w*/*w* UV filters), with an in vitro SPF value of 16 and antioxidant activity at 519.92 ± 0.00 μmol TE g^−1^. With the synergism between natural and synthetic components, the increase in SPF and the negative effect of irradiation on photoprotective activity were observed. Even without showing a photostable behavior, the Formulation F after two hours of UV radiation had an in vitro SPF 39.93% higher than E in the same period. Formulation F protected 17.98% more against UVA radiation (UVA-PF) than E. To evaluate the action of the samples in biological tissue, formulations E and F were tested in human skin. In safety tests, the two samples analyzed did not induce any adverse reactions of irritability, sensitization, phototoxicity or photosensitization. Compared to the E, the F sample demonstrated a 20.59% higher efficiency in vitro and clinical photoprotection. In summary, E and F were considered safe for human topical use and F was statistically superior in both UVA and UVB protection and a greater time was necessary to produce erythematogenic response (phototypes I to III) compared to the sample with only chemical filters. Finally, the negative effects of UV radiation on the skin, such as erythema and photoaging, can be minimized with the antioxidant grape pomace reuse from winemaking in sunscreens.

## Figures and Tables

**Table 1:** Active and bioactive ingredients (% *w*/*w*) used in sunscreens formulations of dried grape pomace (*V. vinifera* L.) and UV filters.

INCI ^1.^	Composition% (*w*/*w*)										
	A	B	C	D	E	F	G	H	I	J	K
*Aqueous phase*											
Ammonium acryloyldimethyltaurate/VP copolymer, trilaureth-4 phosphate, rapeseed oil sorbitol esters, mineral oil and isopropyl palmitate	4.00	4.00	4.00	4.00	4.00	4.00	4.00	4.00	4.00	4.00	4.00
Ammonium acryloyldimethyltaurate vinylpyrrolidone	0.50	0.50	0.50	0.50	0.50	0.50	0.50	0.50	0.50	0.50	0.50
Propylene glycol	5.00	5.00	5.00	5.00	5.00	5.00	5.00	5.00	5.00	5.00	5.00
Ethyl alcohol	2.50	2.50	2.50	2.50	2.50	2.50	2.50	2.50	2.50	2.50	2.50
Disodium EDTA	0.10	0.10	0.10	0.10	0.10	0.10	0.10	0.10	0.10	0.10	0.10
**Grape pomace extract**	1.46	8.54	1.46	8.54	0.00	10.00	5.00	5.00	5.00	5.00	5.00
Water purified q.s.	100.00	100.00	100.00	100.00	100.00	100.00	100.00	100.00	100.00	100.00	100.00
*Oil phase*											
Butylmethoxydibenzoyl methane ^2^	0.73	0.73	4.27	4.27	2.50	2.50	0.00	5.00	2.50	2.50	2.50
Ethylhexyl methoxycinnamate ^3^	1.46	1.46	8.54	8.54	5.00	5.00	0.00	10.00	5.00	5.00	5.00
Ethylhexyl dimethyl PABA ^3^	1.17	1.17	6.83	6.83	4.00	4.00	0.00	8.00	4.00	4.00	4.00
Butyl hydroxy toluene	0.10	0.10	0.10	0.10	0.10	0.10	0.10	0.10	0.10	0.10	0.10
Mixture of phenoxyethanol and parabens others ^4^	0.75	0.75	0.75	0.75	0.75	0.75	0.75	0.75	0.75	0.75	0.75

^1^ International Nomenclature of Cosmetic Ingredient; ^2^ UVA filters (320–400 nm); ^3^ UVB filters (290–320 nm); ^4^ Methylparaben, Ethylparaben, Propylparaben, Butylparaben and Isobutylparaben; pH corrector enough to pH value 5: Sodium hydroxide and Citric acid.

**Table 2:** ANOVA for response surface regression of sun protection factor (Y1), antioxidant activity (Y2), critical wavelength (Y3), UVA transmittance (Y4), UVB transmittance (Y5), and UVA/UVB ratio (Y6) c of the concentration of sunscreens filters (X1), concentration of extract (X2), and time of irradiation (X3).

Source	Y1			Y2			Y3			Y4			Y5			Y6		
	Df	SS	P	Df	SS	P	Df	SS	P	Df	SS	P	Df	SS	P	Df	SS	P
Regression	6	10.26	0.000	4	599.9	0.002	6	6150	0.000	6	11710	0.000	7	8358	0.000	3	0.756	0.000
Linear																		
X1	1	0.89	0.000	1	36.7	0.021	1	3016	0.000	1	1645	0.000	1	1665	0.000	1	0.171	0.000
X2	1	0.32	0.005	1	65.2	0.010	1	15	0.174	1	380	0.009	1	209	0.014	1	0.050	0.000
X3	1	0.90	0.000				1	105	0.002	1	541	0.003	1	484	0.001			
Quadratic																		
X1 × X1	1	0.39	0.002				1	2146	0.000	1	753	0.001	1	942	0.000	1	0.085	0.000
X2 × X2	1	0.07	0.133	1	28.2	0.030				1	183	0.057	1	127	0.048			
X3 × X3	1	0.17	0.029							1	103	0.144	1	82	0.106			
Interaction																		
X1 × X2				1	8.2	0.128	1	39	0.035									
X1 × X3																		
X2 × X3							1	34	0.048				1	98	0.079			
Error	17	0.51		3	5.6		17	128		17	44		16	444		20	0.043	
Total	23	10.77		7	605.5		23	6278		23	12456		23	8802		23	0.799	

**Table 3:** Regression equation and determination coefficients for response surface regression of the sun protection factor (Y1), antioxidant activity (Y2), critical wavelength (Y3), UVA transmittance (Y4), UVB transmittance (Y5), and UVA/UVB ratio (Y6) as functions of the concentration of sunscreen filters (X1), concentration of extract (X2), and time of irradiation (X3).

Regression Equation	^1^ R^2^	^2^ R^2^adj	^3^ R^2^pred
ln(Y1) = 0.341 + 0.2817 × X1 + 0.1258 × X2 − 0.857 × X3 − 0.01409 × X1 × X1 − 0.00579 × X2 × X2 + 0.1795 × X3 × X3	0.9522	0.9354	0.8849
Y2 = 24.35 − 1.017 × X1 + 3.631 × X2 − 0.1831 × X2 × X2 + 0.0861 × X1 × X2	0.9907	0.9783	0.8295
Y3 = 328.35 + 16.112 × X1 + 0.722 × X2 − 4.91 × X3 − 0.9844 × X1 × X1 − 0.1092 × X1 × X2 + 0.477 × X2 × X3	0.9796	0.9723	0.9361
Y4 = 96.51 − 12.11 × X1 − 4.31 × X2 + 20.96 × X3 + 0.617 × X1 × X1 + 0.285 × X2 × X2 − 4.39 × X3 × X3	0.9401	0.9190	0.8581
Y5 = 68.08 − 12.19 × X1 − 3.41 × X2 + 21.78 × X3 + 0.691 × X1 × X1 + 0.238 × X2 × X2 − 3.91 × X3 × X3 − 0.808 × X2 × X3	0.9495	0.9275	0.8681
Y6 = 0.1409 + 0.1159 × X1 − 0.0559 × X3 − 0.006207 × X1 × X1	0.9459	0.9378	0.9082

^1^ Coefficient of determination; ^2^ Adjusted coefficient of determination; ^3^ Predicted coefficient of determination.

**Table 4:** Physical and physicochemical characterization of formulations with and without grape pomace (*V. vinifera* L.) after 24 h of preparation.

Formulation	Concentration (% *w*/*w*)		Organoleptic Characteristics			
	UV Filters ^1^	GP ^2^	Aspect ^3^	Color	Odor ^4^	pH
A	3.37	1.46	N	Light purple	C	5.40
B	3.37	8.54	N	Wine	C	5.46
E	11.50	0.00	N	Yellow	C	5.40
F	11.50	10.00	SI	Dark purple	C	5.49
G	0.00	5.00	N	Wine	C	5.46
I	11.50	5.00	N	Purple	C	5.51
J	11.50	5.00	N	Purple	C	5.45
K	11.50	5.00	N	Purple	C	3.38

^1^ Butylmethoxydibenzoyl methane, ethylhexyl methoxycinnamate and ethylhexyl dimethyl PABA; ^2^ Grape pomace; ^3^ N: Homogeneous and normal consistency and SI: Homogeneous and slightly increased consistency; ^4^ C: Characteristic of the sun filters and/or grape.

**Table 5:** Antioxidant activity of the formulations containing grape pomace (*V. vinifera* L.) by the DoE.

Formulation	% AAO ^1^	Trolox (µmol g^−1^) ^2^
A	26.88 ± 0.02	306.76 ± 0.02
B	41.68 ± 0.01	545.53 ± 0.01
E	12.20 ± 0.00	64.92 ± 0.00
F	40.10 ± 0.00	519.92 ± 0.00
G	36.99 ± 0.00	469.30 ± 0.00
I	32.64 ± 0.00	398.22 ± 0.00
J	30.79 ± 0.01	368.05 ± 0.01
K	30.02 ± 0.01	355.59 ± 0.01

^1^ Antioxidant activity (AAO); ^2^ Standard. The results of antioxidant activity expressed as the mean ± standard deviation (*n* = 3) and linear regression analysis in the 95% confidence interval (*p*-value < 0.05).
